# PTTG1 is involved in TNF‐α‐related hepatocellular carcinoma via the induction of c‐myc

**DOI:** 10.1002/cam4.2473

**Published:** 2019-08-06

**Authors:** Xianyi Lin, Yidong Yang, Yunwei Guo, Huiling Liu, Jie Jiang, Fengping Zheng, Bin Wu

**Affiliations:** ^1^ Department of Gastroenterology The Third Affiliated Hospital of Sun Yat‐Sen University Guangzhou China

**Keywords:** c‐myc, hepatocellular carcinoma, inflammation, pituitary tumor transforming gene 1, proliferation

## Abstract

Hepatocellular carcinoma (HCC) is a malignant disease caused by a variety of factors. However, the genomic and molecular aberrations in HCC are largely unknown. Herein, *pituitary tumor transforming gene 1* (*PTTG1*) was discovered as a potential inflammation‐related oncogene in HCC, and its functions and molecular mechanisms were investigated. mRNA expression microarray, real‐time polymerase chain reaction (PCR), immunohistochemistry, and western blotting analyses revealed that *PTTG1* is upregulated in HCC. Further in vitro and in vivo studies indicated that the proinflammatory cytokine tumor necrosis factor‐α (TNF‐α) induces *PTTG1* expression, and PTTG1 was found to upregulate c‐myc, a well‐known oncogene. Downregulation of PTTG1 reduced c‐myc and proliferating cell nuclear antigen (PCNA) expression and inhibited cell proliferation. Interestingly, inhibition of c‐myc by 10058‐F4 did not affect PTTG1, which suggests that PTTG1 regulates c‐myc expression. Furthermore, PTTG1 expression levels are inversely correlated with HCC patient survival, indicating an independent prognostic biomarker for patients with HCC. Our data demonstrate that PTTG1 is involved in TNF‐α‐related HCC via c‐myc induction and that PTTG1 may be a potential therapeutic target for HCC.

## Graphical abstract

Our data suggest that pituitary tumor transforming gene 1 (PTTG1) plays a vital role in inflammation‐related hepatocellular carcinoma (HCC) via c‐myc induction and that PTTG1 may be a potential therapeutic target for HCC.

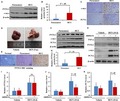

**Abbreviations**
CCl_4_: carbon tetrachloride
DEN: diethylnitrosamine
HCC: hepatocellular carcinoma
PTTG1: pituitary tumor transforming gene 1

## 1 INTRODUCTION

Hepatocellular carcinoma (HCC) is one of the most common cancers and a leading cause of death worldwide.^1^ In the complicated process of HCC development, aberrant expression of oncogenes is frequently observed.^2^ Identifying potential oncogenes and revealing their role in HCC are important. Pituitary tumor transforming gene 1 (PTTG1), a ubiquitously expressed transcription factor, has been verified to be abundantly expressed in many tumors, such as pituitary, thyroid, uterine, ovarian, breast, gastric, and colon cancer, as well as HCC.^3^, ^4^ PTTG1 is involved in the processes of cellular differentiation, apoptosis, DNA damage repair, and angiogenesis.^5^ It is also associated with tumor growth, invasiveness, and aggressive behavior.^6^ PTTG1 can promote the transcription of genes that are directly or indirectly involved in tumorigenesis. Therefore, it is important to elucidate the mechanism of PTTG1 involvement in the process of hepatocellular carcinoma development.

Persistent hepatitis caused by hepatitis virus infection, fatty liver disease, or alcohol abuse is a strong risk factor for hepatocellular carcinogenesis. Persistent hepatitis is associated with inflammation‐induced hepatocyte death and compensatory proliferation, and proinflammatory cytokines play an important role in this pathophysiological process.^7^ Tumor necrosis factor‐α (TNF‐α), an important proinflammatory cytokine involved in HCC, is produced predominantly by macrophages during inflammatory responses.^7^ During hepatocellular carcinogenesis, TNF‐α induces persistent hepatocyte proliferation, increases the malignant transformation of normal hepatocytes and promotes the expression of various oncogenes.^8^ In keratinocytes, TNF‐α leads to the induction of PTTG1 expression.^9^ However, there is limited knowledge of the exact mechanisms of TNF‐α‐induced PTTG1 involvement in hepatocellular carcinogenesis.

A previous study indicated that the well‐known oncogene c‐myc is induced by TNF‐α in colon cancer.^10^ c‐Myc is a powerful transcription factor that is frequently aberrantly amplified in human malignant tumors, and its overexpression promotes cell proliferation, cell cycle progression, and tumor growth.^11^ Evidence based on many studies has revealed that c‐myc overexpression plays an essential role in hepatocellular carcinogenesis as a powerful regulator.^12^ Previous studies identified PTTG1 as a transcription factor that binds to the *c‐myc* promoter and regulates its expression.^13^ In HCC, further investigation is needed to determine the interaction between TNF‐α, PTTG1 and c‐myc.

In this study, a gene assay was performed to measure the expression of mRNAs in human HCC samples, paired HCC paracancerous specimens and normal liver tissues to analyze the possible association of oncogenes in hepatocarcinogenesis. PTTG1 was found to be clearly upregulated in hepatocellular carcinoma. The proinflammatory cytokine TNF‐α‐induced *PTTG1* expression, and PTTG1 upregulated c‐myc. Downregulation of PTTG1 reduced c‐myc and proliferating cell nuclear antigen (PCNA) expression and inhibited cell proliferation. However, depression of c‐myc did not affect PTTG1, suggesting that PTTG1 regulates c‐myc expression. Furthermore, PTTG1 expression levels were inversely correlated with HCC patient survival. These results indicate that PTTG1 plays a vital role in TNF‐α‐related HCC via c‐myc induction and that PTTG1 may be a potential target for HCC therapy.

## 2 MATERIALS AND METHODS

Clinical samples. Normal liver tissue specimens were obtained 2 cm from the parahemangioma edge during resection from five hemangioma patients without hepatitis virus infection. Thirty‐five HCC tissue samples were obtained during operations on HCC patients with hepatitis B virus infection and without any previous therapeutic intervention. Matched paired paracancerous tissues were taken from more than 2 cm from the edge of the tumors. Histological verification of the samples was performed. The study protocol was approved by the Research Ethics Committee of The Third Affiliated Hospital of Sun Yat‐Sen University ([2014]2‐7). Each patient provided written informed consent before inclusion in the study.

The Cancer Genome Atlas (TCGA) data acquisition. The TCGA project provides multimodal data on 374 HCC cases, which can be downloaded from the TCGA website (https://tcga-data.nci.nih.gov/tcga/). The gene expression and clinical data of each case were acquired from the TCGA datasets on February 15, 2019. In the following survival analysis, 31 patients in HCC cohort were excluded because of incomplete clinical information. According to the expression value of *PTTG1*, HCC patients were divided into the low expression and the high expression groups using the median. Because the data from the TCGA are publicly available and acquired by corresponding publication guidelines and data access policies, additional approval by the local ethics committee was not needed.

Cell lines. HepG2 and Huh7 cells, which were confirmed to without mycoplasma contamination, were cultured in Dulbecco's modified Eagle's medium (DMEM) (Gibco BRL, Rockville, MD, USA) containing 10% of fetal bovine serum. For drug administration, TNF‐α (Sigma, St Louis, MO, USA) was dissolved in DMEM and added at different concentrations or time points after the cells reached 90% confluence. For siRNA treatment, HepG2 and Huh7 cells were transfected with PTTG1‐siRNA according to a previously described protocol.^14^ The PTTG1‐siRNA sequences are 5′‐GGGAAUCCAAUCUGUUGCATT‐3′ (sense) and 5′‐UGCAACAGAUUGGAUUCCCTT‐3′ (antisense). The negative control‐siRNA sequences are 5′‐UUCUCCGAACGUGUCACGUTT‐3′ (sense) and 5′‐ACGUGACACGUUCGGAGAATT‐3′ (antisense). 10058‐F4 (Sigma‐Aldrich, St Louis, MO, USA), a small‐molecule c‐myc inhibitor, was dissolved in dimethylsulfoxide (DMSO) and further diluted to 100 μmol/L in DMEM before use as described previously.^15^ 10058‐F4 was added 1 day prior to TNF‐α administration.

Mice. Eight‐week‐old mice (20‐25 g) on a C57BL/6 background and 5‐week‐old male Balb/c athymic nude mice were purchased from Beijing Vital River Laboratory Animal Technology Co., Ltd. (Beijing, China). The mice were housed in microisolator cages under a 12/12 hour dark‐light cycle (lights on at 8:00 am) with food and water ad libitum.

Human Gene Expression Array. Agilent SurePrint G3 microarrays were used as described previously.^14^ Differentially expressed genes were screened using threshold of* P* < .05. Significance of gene expression difference was determined using threshold of fold change >2. The gene expression omnibus (GEO) accession number from the NCBI is GSE67764.

Treatment of mice. Male mice were used in all in vivo studies. The dimethylnitrosamine (DEN)‐induced HCC model and tetrachloromethane (CCl_4_)‐induced cirrhosis model were established following a previously described protocol.^14^ For short‐term studies of DEN‐induced liver injury and inflammation, 8‐week‐old male mice were treated with a single intraperitoneal injection of DEN (100 mg/kg) and killed after 0, 4, 8, 10, or 14 days. The small‐molecule c‐myc inhibitor 10058‐F4 (20 mg/kg) was intraperitoneally injected daily after DEN (100 mg/kg) treatment, and these mice were killed after 10 days. In each group, six mice were used.

Cell viability and colony formation assay. The viability of cells transfected with PTTG1‐siRNA or control‐siRNA was measured using the CCK‐8 assay (Dojindo, Kumamoto, Japan) according to the manufacturer's instructions. Briefly, HepG2 and Huh7 cells were transfected with PTTG1‐siRNA or control‐siRNA in 6‐well plates for 24 hours and then plated in 96‐well plates (1 × 10^3^/well) for 1‐6 days. WST‐8 solution (10 μL) was added to cells in 90 μL culture medium, the cells were incubated at 37°C for 1 hour, and the absorbance was measured at 450 nm. For the colony formation assay, HepG2 and Huh7 cells in 6‐well plates were transfected with control‐siRNA and PTTG1‐siRNA, respectively. After 24 hours of transfection, the cells were collected and seeded (2 × 10^3^/well) in 6‐well plates for 10 days. Colonies containing more than 50 cells were counted after staining with 5% crystal violet. The assay was carried out in triplicate wells in three independent experiments. All experiments were conducted in triplicate wells and repeated three times.

Stable downregulation of PTTG1 with short hairpin RNA (shRNA). To establish a stable Huh7 cell line with PTTG1 downregulation, the cells were transfected with shRNA following a previously reported protocol.^16^ Briefly, for Huh7 cells plated in 6‐well plates, 2.5 μg shRNA plasmid was mixed with 3.75 μL Lipofectamine 3000 and 5 μL P3000 in 250 μL Opti‐MEM per well. The shRNA sequence targeting human PTTG1 (shPTTG1) is 5′‐GGGAGATCTCAAGTTTCAACA‐3′. The shRNA negative control (shNC) plasmid was used as a negative control. Cells stably expressing shRNA were selected in DMEM containing puromycin (2 µg/mL) for 2 weeks. RT‐PCR and western blot analyses were performed to confirm the stable downregulation of PTTG1 in Huh7 cells.

In vivo tumorigenicity assay. Xenograft tumors were established by subcutaneous injection of 5 × 10^6^ Huh7 cells into the right flank of 5‐week‐old male Balb/c athymic nude mice (n = 5 per group). Huh7 cells stably expressed shPTTG1 or shNC. Tumor diameter was measured every 3 for 15 days. Tumor volume (mm^3^) was estimated by measuring the longest and shortest diameters of the tumor and using a previously described equation^14^ Mice were sacrificed 15 days after cell injection. Xenograft tumors were isolated and weighed.

Histological and immunohistochemical staining. Paraffin sections of liver tissue from humans and mice were subjected to H&E and immunohistochemical (IHC) staining as described previously.^14^ Antibodies against PTTG1 (1:400, Abcam, Cambridge, MA; ab26273), c‐myc (1:400, Abcam; ab32072), and PCNA (1:400, Santa Cruz, CA; sc‐56), F4/80 (1:400, Santa Cruz; sc‐377009), and TNF‐α (1:200, Santa Cruz; sc‐52746) were used as primary antibodies for IHC staining. The number of positive cells by IHC was counted per 1,000 cells in each sample.

Western blotting. Total protein extracts were analyzed via western blotting as previously described.^14^ Antibodies against PTTG1 (1:1000, ab26273) and c‐myc (1:1000, ab32072) were obtained from Abcam. Antibodies against PCNA (1:1000, sc‐56) and TNF‐α (1:500, sc‐52746) for western blotting were obtained from Santa Cruz. An antibody against β‐actin (1:7000, A5441) was purchased from Sigma‐Aldrich (St Louis, MO, USA). The band intensity was determined by densitometry, and the normalized expression of each target protein is presented as a ratio to the intensity of the loading control protein.

Semiquantitative RT‐PCR and real‐time polymerase chain reaction. Total RNA isolation and first strand cDNA synthesis were performed according to the manufacturer's instructions.^14^ For semiquantitative RT–PCR, Hot‐start DNA polymerase (Invitrogen, Carlsbad, CA) was used. β‐Actin was employed as an internal control. Real‐time polymerase chain reaction (PCR) was performed using gene‐specific primers and ChamQ SYBR qPCR Master Mix (Vazyme, Nanjing, China) on a real‐time PCR system (Bio‐Rad, CA). RNA was amplified using the following primers: Human *TNF‐α* exon F, 5′‐GTTCCTCAGCCTCTTCTCCT‐3′, and human *TNF‐α* exon R, 5′‐ACAACATGGGCTACAGGCTT‐3′; human *PTTG1* exon F, 5′‐ACCCGTGTGGTTGCTAAGG‐3′, and human *PTTG1* exon R, 5′‐ACGTGGTGTTGAAACTTGAGAT‐3′; and human *c‐myc* exon F, 5′‐GGCTCCTGGCAAAAGGTCA‐3′, and *c‐myc* exon R, 5′‐AGTTGTGCTGATGTGTGGAGA‐3′; and mouse *TNF‐α* exon F, 5′‐TTCTGTCTACTGAACTTCGGGGTGATCGGTCC‐3′, and mouse *TNF‐α* exon R, 5′‐GTATGAGATAGCAAATCGGCTGACGGTGTGGG‐3′. The expression of *β‐actin* was quantified as the internal control using the sense primer 5′‐GTCTTCCCCTCCATCGTG‐3′ and the antisense primer 5′‐AGGGTGAGGATGCCTCTCTT‐3′ for human samples and the sense primer 5′‐GGCTGTATTCCCCTCCATCG‐3′ and the antisense primer 5′‐CCAGTTGGTAACAATGCCATGT‐3′ for mouse samples.

Statistical analysis. All data were analyzed using GraphPad Prism 6.01. The results are presented as the mean ± SD. Student's *t* test or Mann‐Whitney *U* test was used to compare the variables of two groups. The Pearson correlation coefficient was used to evaluate the correlation between *TNF‐α* mRNA expression and *PTTG1* mRNA expression in the clinical samples. The difference in cell viability and tumor growth rate between the two groups of nude mice was determined by repeated measures analysis of variance. A chi‐square test was used to analyze the difference in positivity of PTTG1 staining between human HCC specimens and corresponding paracancerous samples. Correlation between PTTG1 expression and clinicopathological features were estimated by Pearson's Chi‐square test. Kaplan‐Meier survival curve and the log‐rank test were used to evaluate overall survival in relation to expression. Hazard ratio for death associated with PTTG1 expression status was estimated by univariate analyses. Multivariate survival analysis was carried out on all parameters that were found to be significant on univariate level using the Cox regression model. Significant differences were considered if the probability of the difference was <5 in 100 (*P* < .05).

## 3 RESULTS

### 3.1 PTTG1 is involved in hepatocellular carcinoma

**Figure 1:**
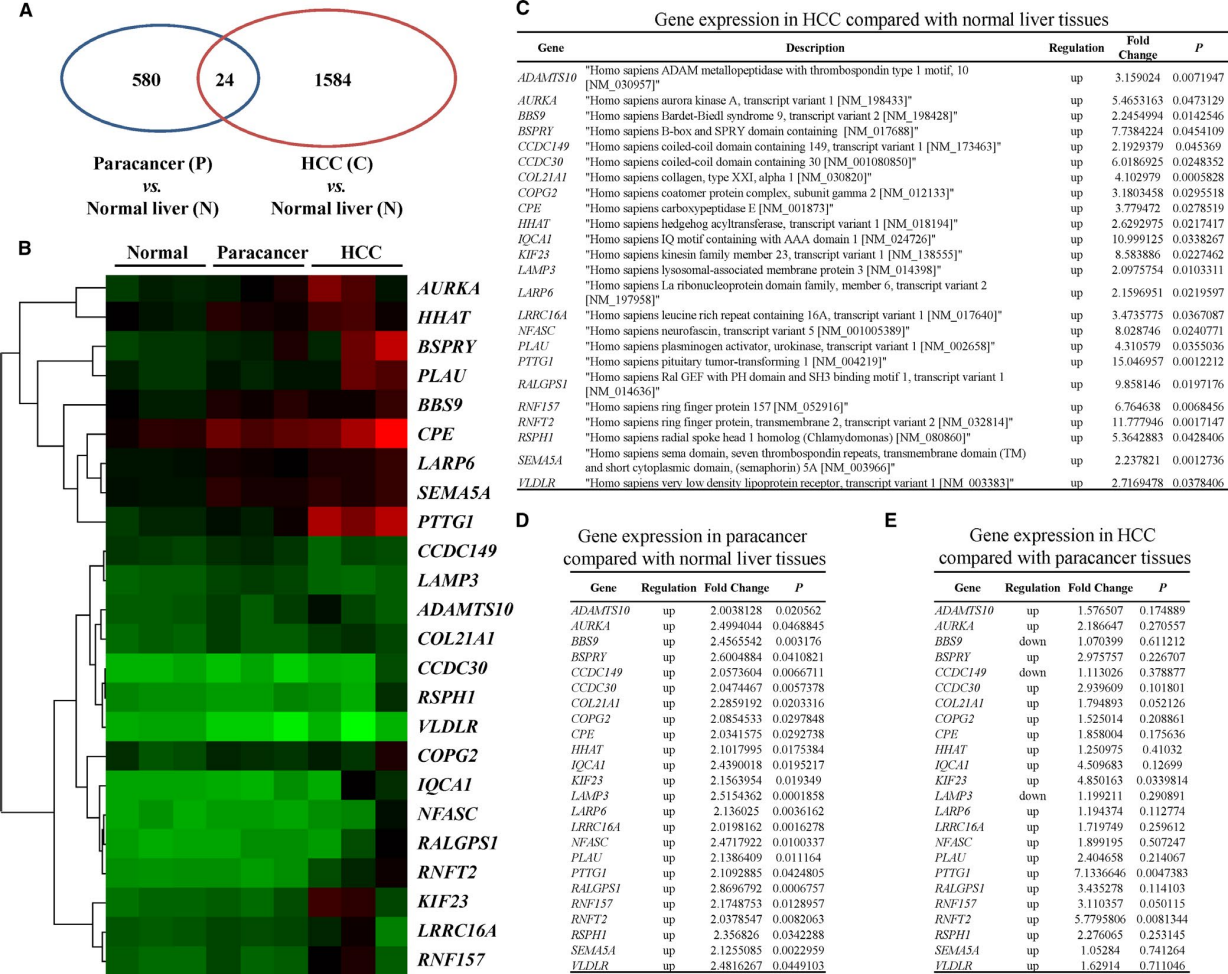
Pituitary tumor‐transforming gene 1 (PTTG1) was specifically upregulated in hepatocellular carcinoma (HCC). mRNA expression profiling was performed to screen for human gene expression alterations in three normal liver tissues and three pairs of paracancer and HCC tissues. A, A total of 580 genes were highly expressed in paracancer tissues compared with normal liver tissues, and 1584 genes were upregulated in HCC tissues compared with normal liver tissues (upregulated more than twofold, *P* < .05). Twenty‐four genes showed markedly higher expression in both paracancer and HCC tissues than in normal liver tissues. B, Two‐dimensional hierarchical clustering results for 24 genes with higher expression in both paracancer and HCC tissues than in normal liver tissues. The fold changes in mRNA levels in paracancer and HCC tissues relative to normal liver tissues are presented by green and red squares showing decreased and increased levels, respectively. C, Expression of 24 genes in HCC tissues compared with normal liver tissues. The ratio represents the expression value in HCC tissues compared with that in normal liver tissues. D, Details of 24 genes expressed in paracancer tissue compared with normal tissue. The ratio represents the expression value in paracancer tissue compared with that in normal liver tissues. E, Expression of 24 genes in HCC tissue compared with paired paracancer tissue. *P* < .05, Student's *t* test

**Figure 2:**
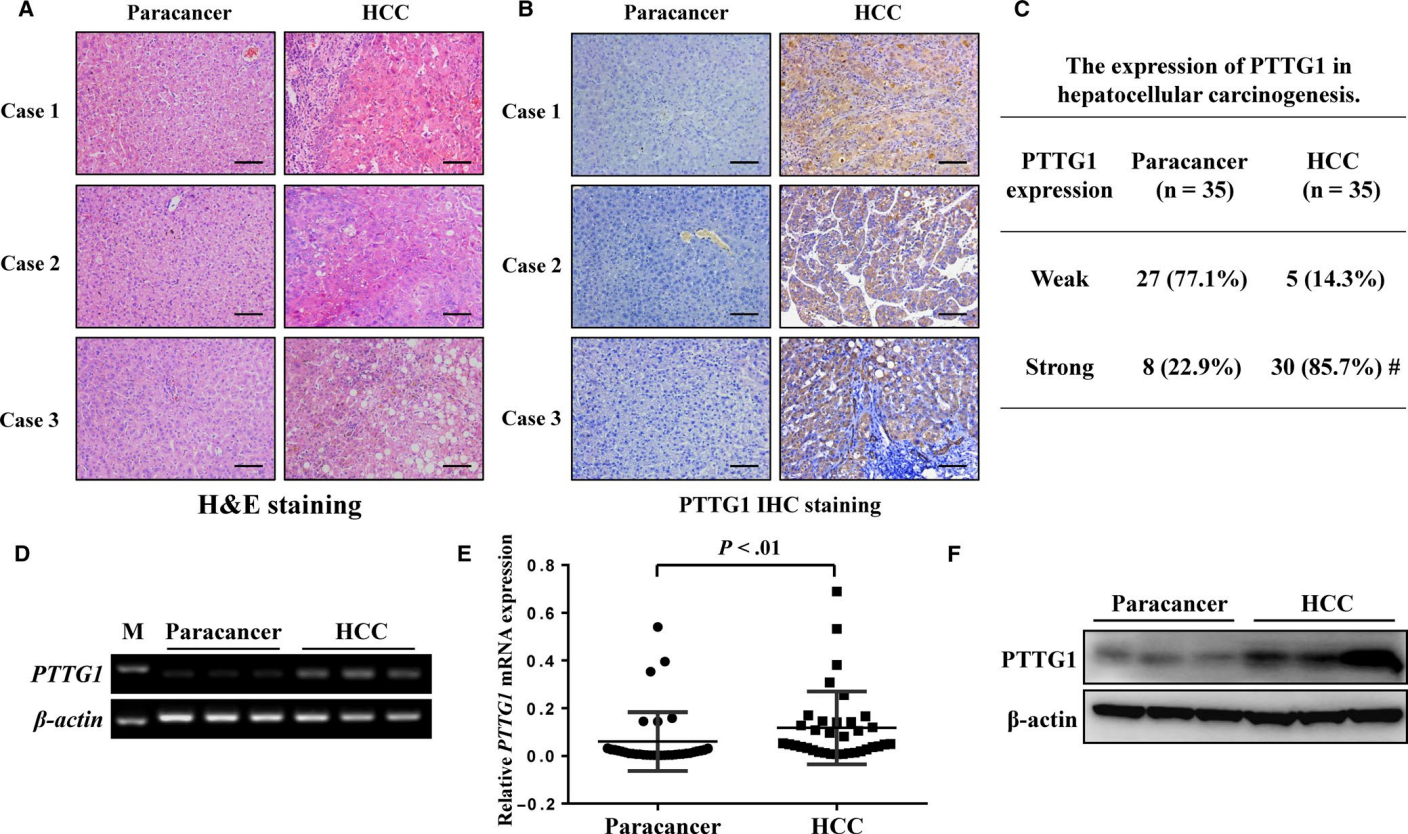
Pituitary tumor‐transforming gene 1 (PTTG1) was involved in hepatocellular carcinoma. A, Representative H&E staining of paracancer tissues and paired hepatocellular carcinoma (HCC) tissues. Scale bar, 100 μm. B, Representative PTTG1 staining of human hepatocellular carcinoma (HCC) and paracancer tissues. Scale bar, 100 μm. C, PTTG1 expression was evaluated by immunohistochemistry. The expression level was evaluated according to the percentage of positive tumor cells (weak, <30%; strong, ≥30%). ^#^ *P* < .05, chi‐square test. D, *PTTG1* mRNA expression in HCC and paracancer tissues determined by polymerase chain reaction (PCR) assay. E, *PTTG1* mRNA levels in HCC and paracancer tissues determined by real‐time PCR. Values are the mean ± SD (n = 35 for the HCC and paracancer groups); Student's *t* test was used. F, PTTG1 protein expression in human HCC and paracancer tissues determined by western blotting

**Figure 3:**
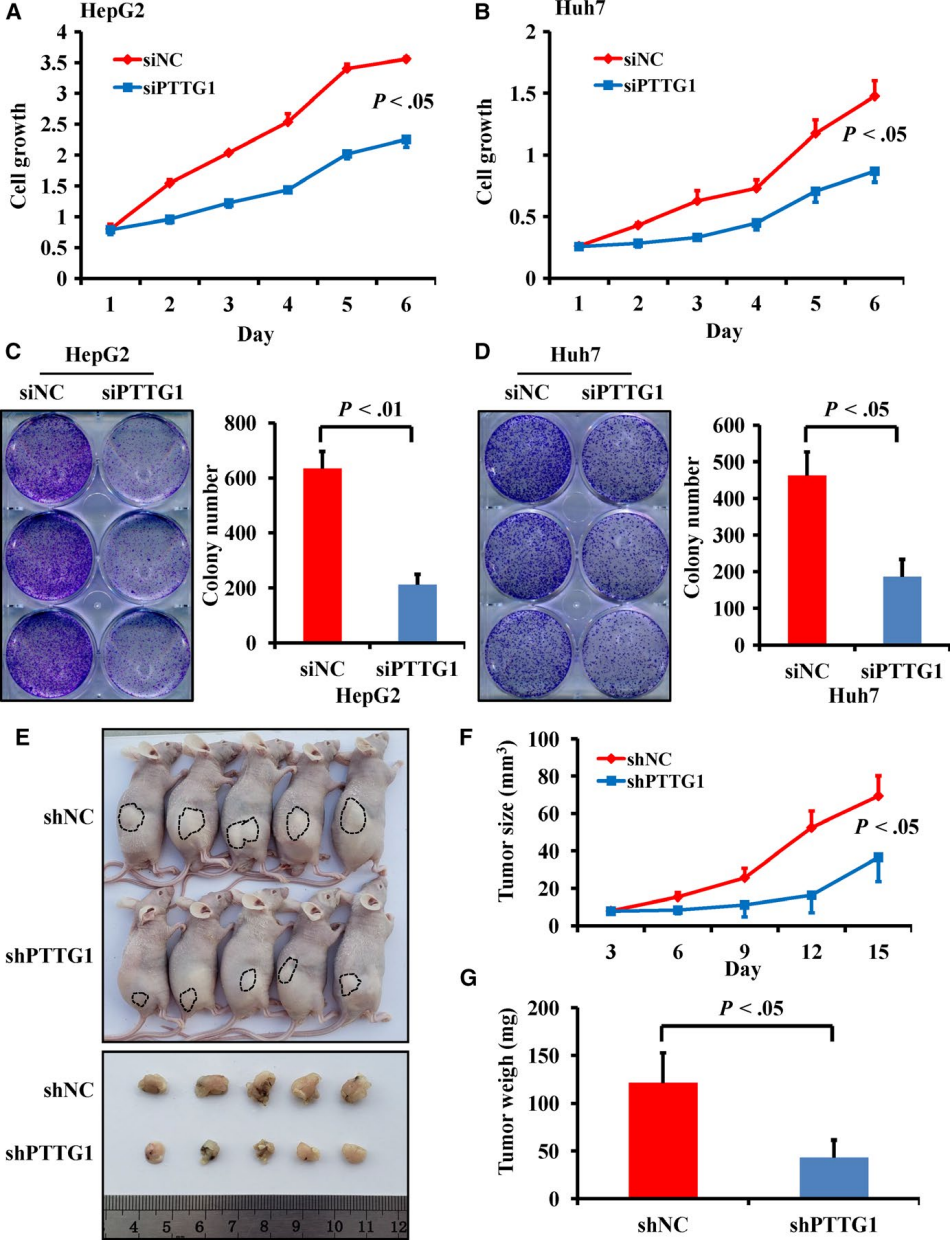
Downregulation of pituitary tumor‐transforming gene 1 (PTTG1) inhibited cell growth in vitro and tumorigenesis in vivo. A, B, Downregulation of PTTG1 by siPTTG1 significantly decreased the viability of HepG2 and Huh7 cells. C, D, siPTTG1‐mediated PTTG1 downregulation suppressed colony formation by HepG2 and Huh7 cells. All values are presented as the mean ± SD of three separate experiments repeated three times. E, Representative images of tumor formation in nude mice subcutaneously inoculated with shNC‐Huh7 or shPTTG1‐Huh7 cells. F, G, Downregulation of PTTG1 by shPTTG1 significantly reduced tumor volume and tumor weight in nude mice. Values are the mean ± SD (n = 5 per group). *P* < .05, Student's *t* test

HCC is generally associated with the aberrant overexpression of oncogenes. To identify potential oncogenes that may be involved in hepatitis‐related HCC, mRNA expression profiling was performed with three normal liver tissues, three HCC tissues characterized by HBV infection and matched paired paracancer tissues. A total of 580 genes were upregulated in paracancer tissues compared with normal liver tissues, and 1584 genes were overexpressed in HCC tissues compared with normal liver tissues (upregulated more than twofold, *P* < .05). In both HCC tissues and paired paracancer tissues, 24 genes were significantly overexpressed compared with normal liver tissues (Figure 1A,B). The detailed expression profiles of these 24 genes are shown in Figure 1C‐E. Among these 24 genes, *PTTG1* exhibited the highest expression in HCC tissues compared with normal liver tissues (upregulated more than 15‐fold, *P* < .01). PTTG1 plays a meaningful role in hepatocellular carcinoma. Furthermore, PTTG1 expression in 35 paired HCC and adjacent paracancer tissues was confirmed by immunohistochemistry staining. Human specimens were verified by histology (Figure 2A). A small amount of PTTG1 expression was observed in paracancer tissues. A higher PTTG1‐positive staining rate in the cytoplasm was found in the HCC samples (85.7%) than in the paired paracancer samples (22.9%) (Figure 2B,C). *PTTG1* expression at both the mRNA and protein levels was examined by real‐time PCR and western blotting, and the results showed that *PTTG1* was overexpressed in HCC samples compared with corresponding paracancer tissues (Figure 2D‐F). The overexpression of PTTG1 in human HCC tissues suggests that PTTG1 may have a tumor‐promoting function in hepatocarcinogenesis. To prove this hypothesis, the role of PTTG1 in cell proliferation was evaluated by conducting cell viability assays and colony formation assays in HepG2 and Huh7 cell lines transfected with PTTG1‐specific siRNA (siPTTG1) to downregulate *PTTG1*. PTTG1 downregulation significantly suppressed the viability of HepG2 and Huh7 cells compared with negative control (siNC)‐siRNA transfection (Figure 3A,B). Similarly, siPTTG1‐transfected HepG2 and Huh7 cells formed significantly fewer colonies than siNC‐transfected HepG2 and Huh7 cells (Figure 3C,D). To further investigate the in vivo protumorigenic activity of PTTG1, *PTTG1* was stably knocked down in Huh7 cells by shPTTG1 transfection. The shNC‐transfected and shPTTG1‐transfected Huh7 cells were injected subcutaneously into the dorsal right flank of nude mice (n = 5 per group) (Figure 3E). The tumor growth rate of shPTTG1‐treated Huh7 cells was significantly slower than that of shNC‐transfected cells (*P* < .05) (Figure 3F). Fifteen days after injection, the mice were sacrificed, and the xenograft tumors were excised; the tumor weight in the shPTTG1‐transfected group was reduced by more than 40% compared with that in the negative control group (*P* < .05) (Figure 3G). These results implied that PTTG1 may be involved in HCC by promoting cell proliferation.

### 3.2 The proinflammatory factor TNF‐α facilitates PTTG1 expression

**Figure 4:**
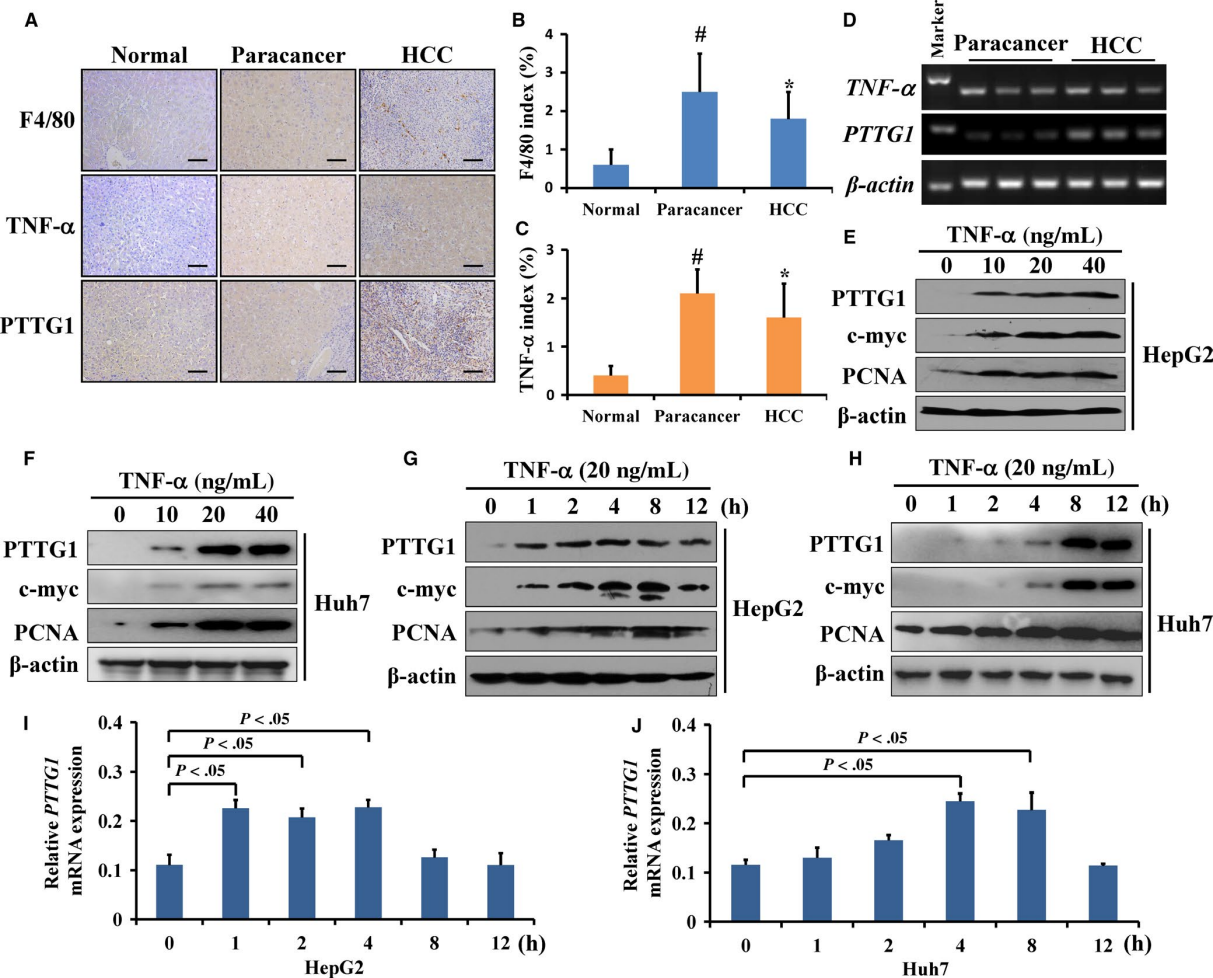
The proinflammatory mediator TNF‐α enhanced pituitary tumor‐transforming gene 1 (PTTG1) expression. A, F4/80 staining for macrophages (top), TNF‐α staining (middle), and PTTG1 staining (bottom) of human normal liver, hepatocellular carcinoma (HCC), and paracancer tissues. Scale bar, 100 μm. B, C, The index of F4/80 (top)‐ and TNF‐α (bottom)‐positive cells in the liver was determined by counting 1000 cells/sample. Values are the mean ± SD (n = 5 per group); #*P* < .05 compared with normal liver tissues, *P < .05 compared with normal liver tissues, Student's *t* test. D, *TNF‐α* and *PTTG1* mRNA expression in HCC and paracancer tissues determined by PCR assay. E, F, HepG2 and Huh7 cells were treated with TNF‐α (0, 10, 20, or 40 ng/mL) for 8 h, and the expression of ARRB1 was analyzed by western blotting. G, H, Samples were collected at 0, 1, 2, 4, 8, and 12 h after HepG2 and Huh7 cells were treated with 20 ng/mL TNF‐α. The expression of PTTG1, c‐myc, and PCNA was analyzed by western blotting. I, J The mRNA expression of *c‐myc* was analyzed by real‐time PCR at 0, 1, 2, 4, 8, and 12 h after HepG2 and Huh7 cells were treated with 20 ng/mL TNF‐α. The experiment was repeated three times. *P* < .05, Student's *t* test

**Figure 5:**
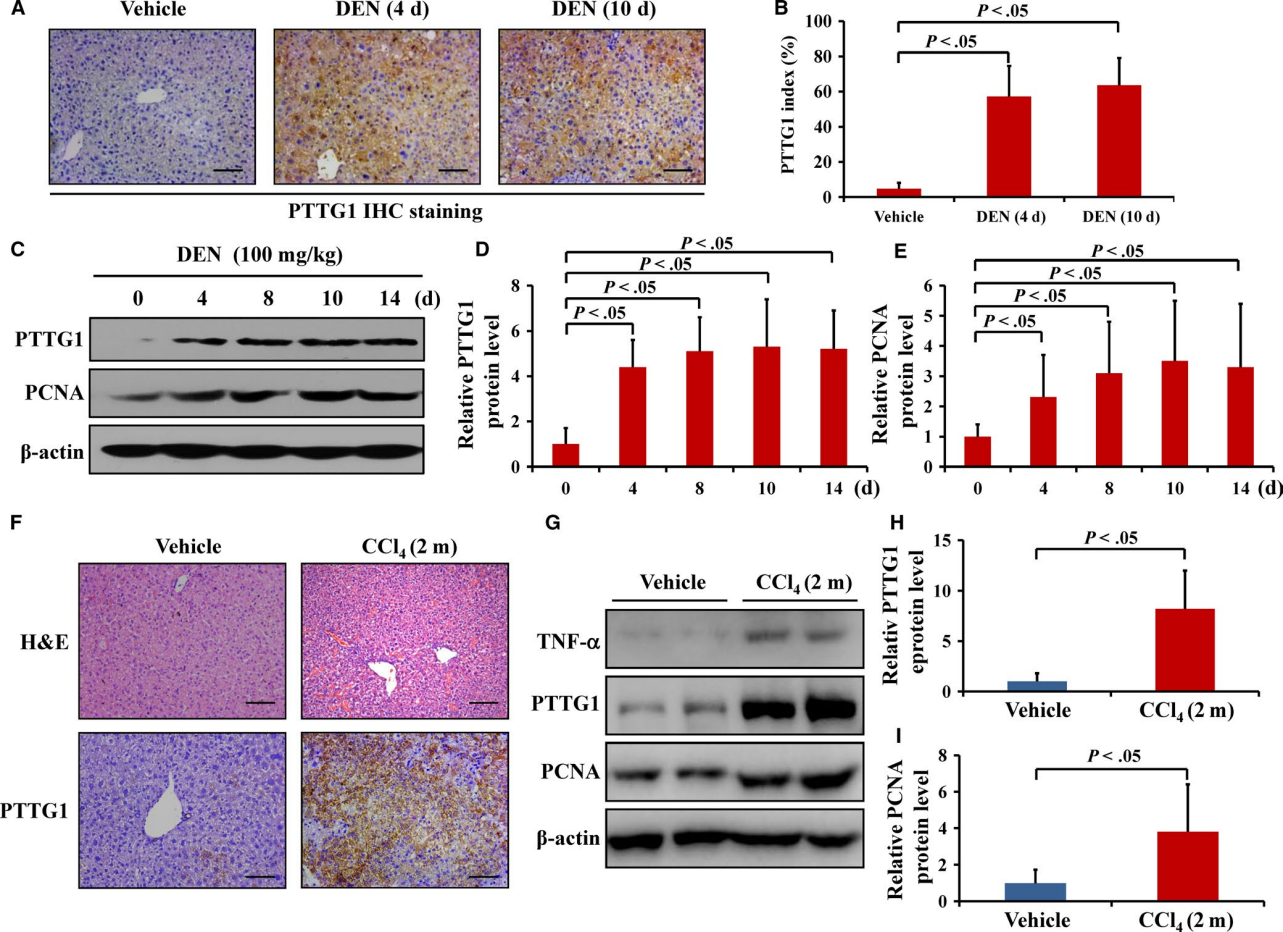
Pituitary tumor‐transforming gene 1 (PTTG1) was involved in inflammation‐mediated hepatocellular carcinogenesis in mice. A, Liver tissues were harvested from mice at 0, 4, and 10 days after intraperitoneal injection of diethylnitrosamine (DEN) (100 mg/kg). Representative expression of PTTG1 by immunohistochemical staining at the indicated time point. Scale bar, 100 μm. B, The index of PTTG1‐positive cells in the liver was measured by counting 1000 cells/sample. Values are the mean ± SD (n = 6 per group); *P* < .05, Student's *t* test. C‐E, PTTG1 and PCNA protein expression in mouse livers at 0, 4, 8, 10, and 12 days after intraperitoneal injection of DEN (100 mg/kg) as determined by western blotting assay. Values are the mean ± SD (n = 6 per group). *P* < .05, Student's *t* test. F, Representative images of H&E (top) and PTTG1 (bottom) staining of CCL_4_‐induced liver cirrhosis. Scale bar, 100 μm. G‐I, The expression of TNF‐α, PTTG1, and PCNA in liver tissues from mice treated with CCl_4_ for 2 mo was evaluated by western blotting, and β‐actin served as a loading control (n = 6 per group). Student's *t* test was used

TNF‐α, which is mainly secreted by macrophages in the liver, plays an important role in HCC. Our previous data showed PTTG1 overexpression in human HCC tissues with HBV infection. However, it was unclear whether TNF‐α induces PTTG1 expression directly in HCC. In this study, F4/80 staining by IHC was used to evaluate macrophage infiltration. In both paracancer tissues and HCC samples, a large amount of F4/80 staining was found. However, little F4/80 staining was observed in normal liver tissues (Figure 4A). The proinflammatory cytokine TNF‐α, which is mainly secreted by macrophages, was markedly induced in both paracancer and HCC tissues compared to normal liver samples (Figure 4B,C), while there was no significant difference in *TNF‐α* mRNA levels between paracancer and HCC tissues (*P* = .94) (Figure S1A). In HCC tissues, *TNF‐α* mRNA expression was positively correlated with *PTTG1* mRNA expression (*R* ^2^ = 0.62, *P* < .01) (Figure 4D; Figure S1B), while in paracancer tissues, *TNF‐α* mRNA levels had no significant correlation with *PTTG1* mRNA levels (*R* ^2^ = 0.02, *P* = .52) (Figure S1C). HepG2 and Huh7 cells were treated with TNF‐α and PTTG1 protein and mRNA levels were upregulated in a dose‐ and time‐dependent manner (Figure 4E‐J). Furthermore, we established two mouse models of liver injury‐induced inflammation by administration of diethylnitrosamine (DEN) for 10 days or tetrachloromethane (CCl_4_) for 2 months. A previous study showed that after DEN or CCl_4_ treatment, the hepatic inflammatory mediator TNF‐α is dramatically upregulated.^14^ Herein, *TNF‐α* mRNA levels in liver tissues were significantly upregulated in the DEN‐ or CCl_4_‐treated groups compared to the control group (Figure S2A, B). Meanwhile, the protein levels of PTTG1 in mouse hepatic tissues were significantly upregulated after DEN (Figure 5A‐E) or CCl_4_ treatment (Figure 5F‐I). Altogether, these results suggested that PTTG1 expression can be induced by TNF‐α in vitro and in vivo.

### 3.3 PTTG1 is involved in hepatocellular proliferation via c‐myc induction

**Figure 6:**
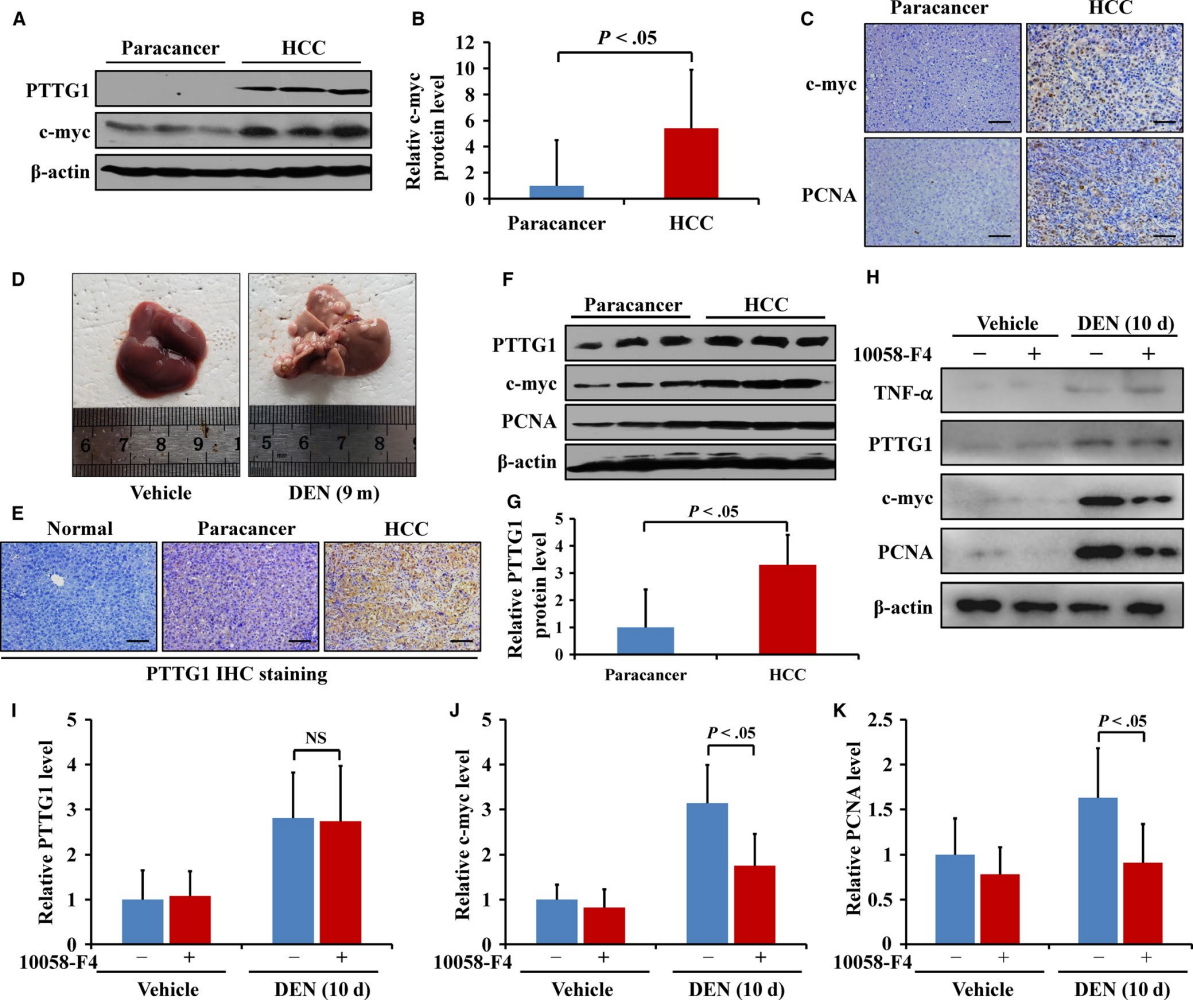
Pituitary tumor‐transforming gene 1 (PTTG1) promoted hepatocellular proliferation through c‐myc induction. A, PTTG1 and c‐myc protein expression in human hepatocellular carcinoma (HCC) and paracancer tissues determined by western blotting. B, Quantitative analysis of c‐myc/β‐actin by densitometry scanning of western blots. (n = 6 for the HCC and paracancer groups). C, Representative images of c‐myc (top) and PCNA (bottom) staining in HCC and paired paracancer tissues. Scale bar, 100 μm. D, C57BL/6 mice received a single intraperitoneal injection of diethylnitrosamine (DEN) (15 mg/kg), and livers were harvested at 9 mo. Photograph of DEN‐induced liver tumors at 9 mo. E, Representative PTTG1 staining of DEN‐induced liver tumors in mice. Scale bar, 100 μm. F, G, The expression of PTTG1, c‐myc, and PCNA was analyzed by western blotting in mouse liver tumor tissues and pair paracancer tissues. Values are the mean ± SD. H‐K, The expression of TNF‐α, PTTG1, c‐myc, and PCNA was analyzed by western blotting in mouse livers 10 d after a single intraperitoneal injection of DEN (100 mg/kg). DEN‐induced TNF‐α, PTTG1, c‐myc, and PCNA; however, a single intraperitoneal injection of DEN (100 mg/kg) combined with daily c‐myc inhibitor 10058‐F4 treatment (20 mg/kg) by intraperitoneal administration for 10 d repressed the expression of c‐myc and PCNA but did not affect PTTG1 expression. (n = 6 in each group). All values are the mean ± SD. Student's *t* test was used. NS: not significant

Previous reports have shown that PTTG1 can induce *c‐myc* expression. Our study showed that the simultaneous upregulation of c‐myc, a well‐known oncogene, and PCNA, a widely recognized proliferation marker, in human HCC tissues with PTTG1 overexpression (Figure 6A‐C). To further elucidate the role of PTTG1 in HCC, this type of cancer was induced with DEN in male C57BL/6 mice. Tumors were evaluated 9 months after DEN injection (Figure 6D). Immunohistochemical staining and western blotting showed that the protein levels of PTTG1 and c‐myc were markedly increased in HCC tissues compared to paracancer tissues (Figure 6E‐G). DEN‐induced extensive hepatocyte apoptosis and subsequent compensatory proliferation, which are essential for hepatocellular carcinoma in mice.^7^ A subsequent study in mice with short‐term DEN treatment mice was performed to determine whether PTTG1 contributes to hepatocellular carcinogenesis by enhancing compensatory hepatocyte proliferation. In these mice, PTTG1 and c‐myc expressions were upregulated, and compensatory hepatocellular proliferation was increased. However, in 10058‐F4‐treated mice, c‐myc expression was downregulated, and compensatory proliferation was repressed without any change in PTTG1 expression (Figure 6H‐K). These results suggested that PTTG1 promotes hepatocellular proliferation through the induction of c‐myc, while c‐myc does not affect *PTTG1* expression in vivo.

### 3.4 Inflammation‐induced HCC depends on PTTG1/c‐myc induction

**Figure 7:**
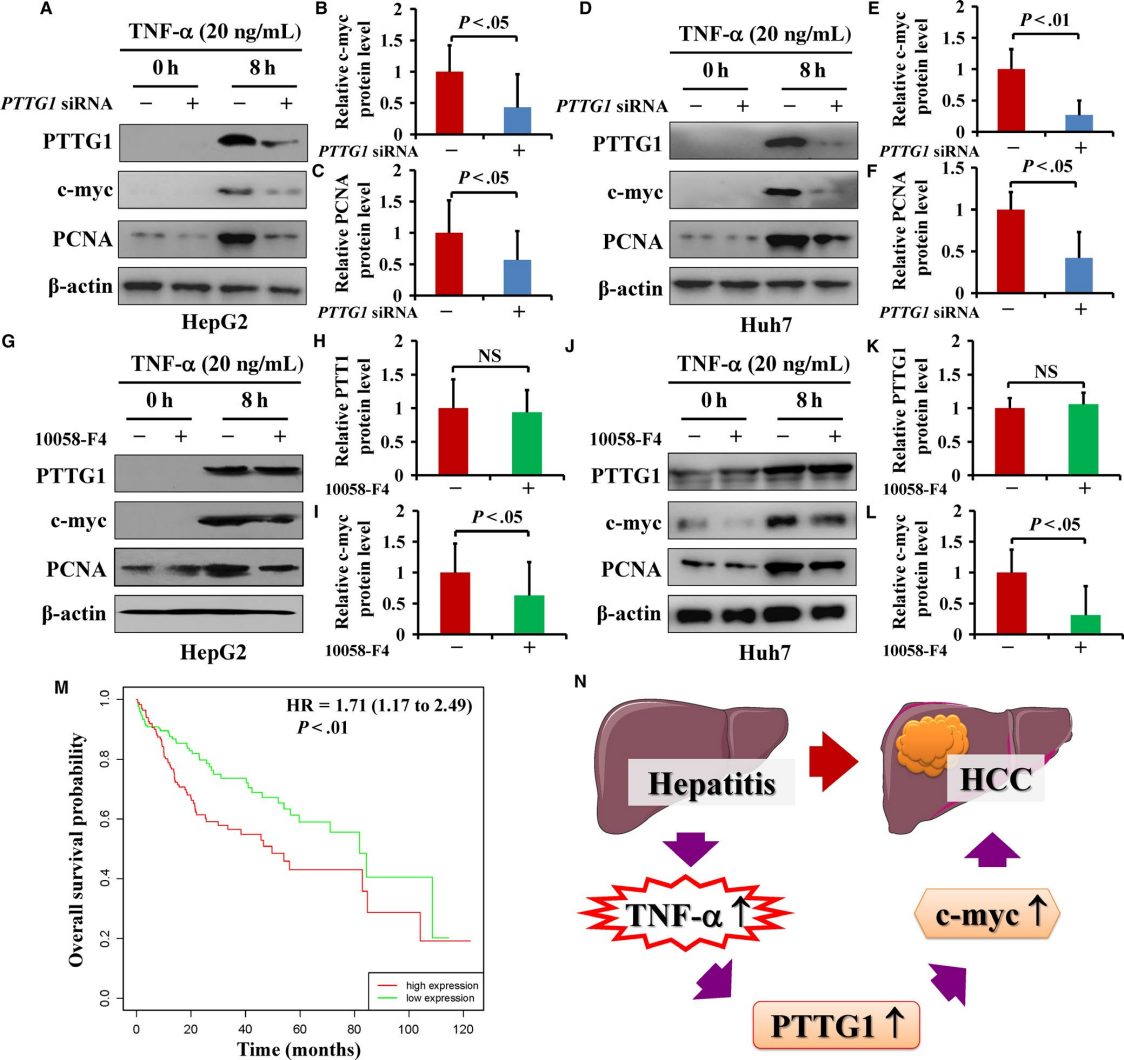
Pituitary tumor‐transforming gene 1 (PTTG1) promoted c‐myc expression and was associated with the prognosis of hepatocellular carcinoma (HCC) patients. A‐F, After 48 h of PTTG1‐siRNA administration, HepG2 and Huh7 cells were treated with TNF‐α (20 ng/mL) for 8 h, and the expression of PTTG1, c‐myc, and PCNA was analyzed by western blotting. PTTG1 expression was downregulated by PTTG1‐siRNA. PTTG1 downregulation significantly reduced c‐myc and PCNA production in HepG2 and Huh7 cells. G‐L, HepG2 and Huh7 cells were treated with the c‐myc inhibitor 10058‐F4 after TNF‐α (20 ng/mL) administration. The c‐myc inhibitor clearly inhibited TNF‐α‐induced c‐myc expression and repressed PCNA expression but had no effect on PTTG1 expression. All values are the mean ± SD of three separate experiments performed in triplicate. Student's *t* test: NS, not significant. M, Kaplan‐Meier plots of OS and PTTG1 expression using the online program Kaplan‐Meier Plotter. Kaplan‐Meier analysis showed that HCC patients with high expression of PTTG1 had markedly worse overall survival than those with low PTTG1 expression [HR = 1.71 (1.17‐2.49) *P* < .01]. N, Schematic diagram of PTTG1 involvement in HCC. TNF‐α‐induced malignant hepatocellular proliferation depends on PTTG1‐mediated c‐myc induction in HCC

Although we verified that c‐myc participates in PTTG1‐mediated proliferation in liver carcinogenesis and others have demonstrated that c‐myc is a main downstream target of PTTG1, it remains unknown whether c‐myc is an important target of PTTG1 in HCC. In this study, *PTTG1* mRNA expression was significantly downregulated in HepG2 or Huh7 cells treated with siRNA for 48 hours (Figure S3A,B). Meanwhile, *c‐myc* mRNA expression was detected and results showed that PTTG1 downregulation by siRNA significantly reduced *c‐myc* mRNA expression in HepG2 and Huh7 cells (Figure S3C,D). We further performed analyses at a single time point (8 hours) after treatment with TNF‐α (20 ng/mL) to analyze the influence of PTTG1 and c‐myc upon exposure to stimuli. As expected, downregulating *PTTG1* by siRNA in the HepG2 cell line obviously decreased the expression of *PTTG1*, as well as that of c‐myc and PCNA (Figure 7A‐C; Figure S4A). Similar results were observed in the Huh7 cell line under the same treatment conditions (Figure 7D‐F; Figure S4B). Furthermore, we inhibited c‐myc expression by 10058‐F4 in HepG2 and Huh7 cells and found that c‐myc levels were decreased and that PCNA expression was inhibited, but there was no effect on the expression of PTTG1 (Figure 7G‐L; Figure S4C, D). The data suggested that c‐myc, a downstream target of PTTG1, can regulate cell proliferation upon stimulation by TNF‐α.

### 3.5 Overexpression of PTTG1 is associated with poor prognosis of patients with HCC

Finally, the correlation between PTTG1 expression status and clinicopathologic features of 343 HCCs was further evaluated using TCGA datasets.^17^ As shown in Table 1, no correlation was observed between PTTG1 overexpression and patient's gender and age. However, overexpression of PTTG1 was positively associated with more advanced tumor size, tumor grade, and TNM stage (*P* < .01), which is consistent with the role of PTTG1 on tumor proliferation. Kaplan‐Meier survival analysis showed that high expression of PTTG1 was associated with markedly worse overall survival compared with low PTTG1 expression in HCC patients (Figure 7M). In univariate Cox regression analysis, overexpression of PTTG1 (*P* < .01), large tumor size (*P* < .01) and advanced TNM stage (*P* < .01) were significant negative prognostic factors for overall survival in HCC patients (Table 2). After the adjustment for potential confounding factors, multivariate Cox regression analysis showed that PTTG1 overexpression was an independent predictor of poorer survival of patients with HCC (HR, 1.49; 95% CI 1.01 to 2.18; *P* = .04). Taken together, these results indicated that TNF‐α‐induced PTTG1 contributes to hepatocellular carcinogenesis by mediating c‐myc expression (Figure 7N).

**Table 1:** Relationship between PTTG1 expression and clinicopathological features in 343 HCC cases

Variable	Low expression (n = 172)	Percent	High expression (n = 171)	Percent	*P* value
Mean age, years ± SD	59.8 ± 13.8		57.6 ± 13.2		.14
Gender					
F	50	45.9%	59	54.1%	.30
M	122	52.1%	112	47.9%	
Tumor size					<.01
≤5 cm	143	55.6%	114	44.4%	
>5 cm	29	33.7%	57	66.3%	
Tumor grade					<.01
G1	31	68.9%	14	31.1%	
G2	98	58.3%	70	41.7%	
G3 and G4	43	33.1%	87	66.9%	
TNM stage					<.01
I	105	62.1%	64	37.9%	
II	36	42.9%	48	57.1%	
III and IV	31	34.4%	59	65.6%	

**Table 2:** Cox regression analysis of potential poor prognostic factors for patients with HCC

Variables	Univariable analysis		Multivariable analysis	
	HR (95% CI)	*P* value	HR (95% CI)	*P* value
Age (<60 vs ≥ 60 years)	1.29 (0.89‐1.87)	.18	—	—
Gender (female vs male)	0.24 (0.54‐1.17)	.24	—	—
Tumor size (≤5 cm vs > 5 cm)	2.46 (1.68‐3.58)	<.01	1.55 (0.21‐11.27)	.67
Tumor grade (G1 and G2 vs G3 and G4)	1.12 (0.77‐1.64)	.55	—	—
TNM stage (I and II vs III and IV)	2.44 (1.67‐3.55)	＜.01	1.47 (0.20‐10.66)	.70
PTTG expression (low vs high)	1.71 (1.17‐2.49)	<.01	1.49 (1.01‐2.18)	.04

## 4 DISCUSSION

Various factors are involved in the initiation, development, and progression of HCC, but the exact mechanisms are still unclear. The causal relationship between aberrant expression of specific oncogenes and HCC has been well documented in epidemiological and functional studies.^18^ In this study, mRNA expression profiling was performed to screen for potential oncogenes in normal liver tissues, HCC tissues and paired HCC paracancer tissues. The overlapping genes upregulated in both cancer and paracancer tissues compared with normal tissue may be potential oncogenes.^19^ *PTTG1* exhibited the highest expression in HCC samples compared to normal liver tissues. To further confirm the overexpression of PTTG1 in HCC, real‐time PCR, western blotting and IHC were performed, and the results showed that PTTG1 was significantly upregulated in human HCC and DEN‐induced HCC mouse tissues. Consistent with previous studies indicating that the overexpression of PTTG1 is involved in HCC development,^20^ our results suggest that PTTG1 is a potential oncogene in HCC.

PTTG1, a securin protein that acts as a sister‐chromatid separation inhibitor, is originally isolated from rat pituitary tumor cells.^21^ In addition to its securin function,^22^ PTTG1 has been reported to facilitate malignant cell proliferation and promote tumor growth.^23^, ^24^ The mechanism by which PTTG1 is involved in cell proliferation includes inducing the upregulation of cyclin B1, cyclin D3, and CDK1, inhibition of SMAD3, separase cleavage and aurora kinase A activity, as well as repression of p21 expression.^24^, ^25^, ^26^, ^27^, ^28^ In this study, we found that downregulation of PTTG1 could suppress the proliferation of two cell lines in vitro and tumorigenesis in nude mice in vivo. The above series of in vitro and in vivo functional experiments revealed that PTTG1 promotes cell proliferation in HCC. These results are consistent with a previous report that PTTG1 is upregulated in HCC and promotes cell proliferation.^29^

Aberrant expression of PTTG1 has been verified in pituitary, thyroid, uterine, ovarian, breast, gastric, and colon cancer.^3^, ^4^ Previous study also demonstrated that PTTG1 is overexpressed in HCC.^29^ PTTG1 overexpression can be induced by cancer‐associated fibroblasts,^30^ E2F1,^31^ Oct‐1,^32^ estrogen, and insulin.^33^ HBx can induce the abnormal accumulation of PTTG1 by inhibiting the ubiquitination of PTTG1 in HBV‐infected liver.^34^ However, the exact mechanism of PTTG1 overexpression in HCC was still unclear. Hepatitis B or C virus infection, alcohol abuse or fatty liver disease may cause persistent death of hepatocytes, induce immune reactions and give rise to chronic inflammation of the liver. Previous studies have demonstrated that inflammation facilitates hepatocellular carcinogenesis and progression through the secretion of proinflammatory cytokines by a variety of cell types. Accumulating evidence suggests that proinflammatory cytokines, including TNF‐α, IL‐6, IL‐1α, IL‐1β, and IFN‐γ, play a vital role in modulating autonomous growth signaling, which influences HCC growth and invasion, and TNF‐α is one of the most critical proinflammatory cytokines.^7^ TNF‐α plays a vital role in cancer by promoting the aberrant expression of a variety of genes through signaling pathway activation.^35^ The proinflammatory cytokine TNF‐α, which is mainly secreted by macrophages, can induce PTTG1 mRNA and protein expression in a dose‐dependent manner in keratinocytes.^9^ Our study showed that TNF‐α was upregulated in both HCC and paracancer tissues compared with normal liver tissues. In HCC tissues, but not paracancer tissues, the expression of TNF‐α was positively correlated with PTTG1 expression. In *vitro* studies further confirmed that TNF‐α promoted PTTG1 expression in a dose‐ and time‐dependent manner. The distinct effect of TNF‐α on PTTG1 expression between HCC tissue and corresponding paracancer tissue may contribute to different cellular responses to TNF‐α0.^36^ TNF‐α triggers apoptosis and growth arrest in normal cells but promotes the proliferation of cancer cells.^37^ Persistent liver inflammation and compensatory hepatocyte proliferation are induced after DEN or CCl_4_ administration to mice.^38^, ^39^ Proinflammatory cytokines, including TNF‐α are upregulated after 10 days of DEN treatment or 2 months of CCl_4_ treatment in mice.^14^, ^40^, ^41^ Our data showed that the administration of DEN or CCl_4_ significantly increased *TNF‐α* expression and induced PTTG1 expression in mouse livers. The results indicated that PTTG1 is a downstream target in the response to inflammation in mice and that TNF‐α induces *PTTG1* expression in hepatocytes.

In addition to the mechanism mentioned above, PTTG1 has also been reported to promote cancer cell proliferation by inducing the upregulation of c‐myc.^13^ PTTG1 promotes *c‐myc* transcription by directly interacting with its promoter, which links PTTG1 to a functional pathway involved in cell proliferation. In this study, PTTG1 overexpression was accompanied by the upregulation of c‐myc and PCNA in human HCC samples. A single intraperitoneal injection of DEN (15 mg/kg) in 15‐day‐old male mice induces hepatocellular carcinogenesis, providing an animal model for HCC research in vivo.^7^, ^42^ In DEN‐induced HCC mouse tissues, the results showed that PTTG1 was upregulated, accompanied by increased c‐myc and PCNA expression. To elucidate whether *c‐myc* is a downstream target gene of PTTG1 in HCC, the c‐myc inhibitor 10058‐F4 was intraperitoneally injected daily into mice after DEN treatment, and the mice were killed after 10 days. 10058‐F4 can induce cell cycle arrest and downregulate c‐myc expression.^15^ The results showed that 10058‐F4 repressed c‐myc protein levels but did not affect PTTG1 expression. HepG2 and Huh7 cells treated with PTTG1‐siRNA showed the downregulation of PTTG1, c‐myc and PCNA expression. 10058‐F4 treatment of HepG2 and Huh7 cells led to the downregulation of c‐myc and PCNA expression without affecting PTTG1 expression.

Prognosis of HCC patients vary greatly. Important clinical prognostic indicators of disease outcome include tumor grade and TNM staging. However, some HCC patients with low‐grade tumors or low TNM stages still have poor outcomes. Therefore, novel prognostic biomarkers are needed to explore for clinical outcome assessment. Previous studies have reported that PTTG1 is associated with aggressive disease and poor prognosis in a variety of malignant tumors, including HCC.^20^, ^28^, ^43^ In this regard, we further examined the influence of PTTG1 overexpression on HCC patients’ clinical outcomes. In agreement with the above studies, a clinical association evaluation showed that overexpression of PTTG1 was associated significantly with more advanced tumor size, tumor grade, and TNM stage. In both univariate and multivariate cox regression analyses, overexpression of PTTG1 was correlated with poor prognosis, suggesting that PTTG1 overexpression could be regarded as an independent new prognostic marker for HCC.

In summary, PTTG1 is upregulated in HCC and contributes to TNF‐α signaling. PTTG1 upregulates c‐myc. Downregulation of PTTG1 reduces c‐myc expression and inhibits cell proliferation, while inhibition of c‐myc does not affect PTTG1 expression, suggesting that c‐myc is a downstream target of PTTG1. Moreover, PTTG1 may serve as an independent prognostic biomarker for patients with HCC. Taken together, these results indicate that PTTG1 plays a vital role in TNF‐α‐related HCC via c‐myc induction and that PTTG1 may be a potential therapeutic target in hepatocellular carcinogenesis.

## CONFLICT OF INTEREST

The authors declare that they have no competing interests.

## Data Availability

The datasets used and/or analyzed during the current study are available from the corresponding author on reasonable request.
