# Supplementary material for: PTTG1 is involved in TNF‐α‐related hepatocellular carcinoma via the induction of c‐myc
Source: Cancer Med. 2019 Aug 6;8(12):5702–15. doi: 10.1002/cam4.2473 (PMC6745867; doi:10.1002/cam4.2473)
Supplement: Supplementary file 1 [file CAM4-8-5702-s001.pdf]

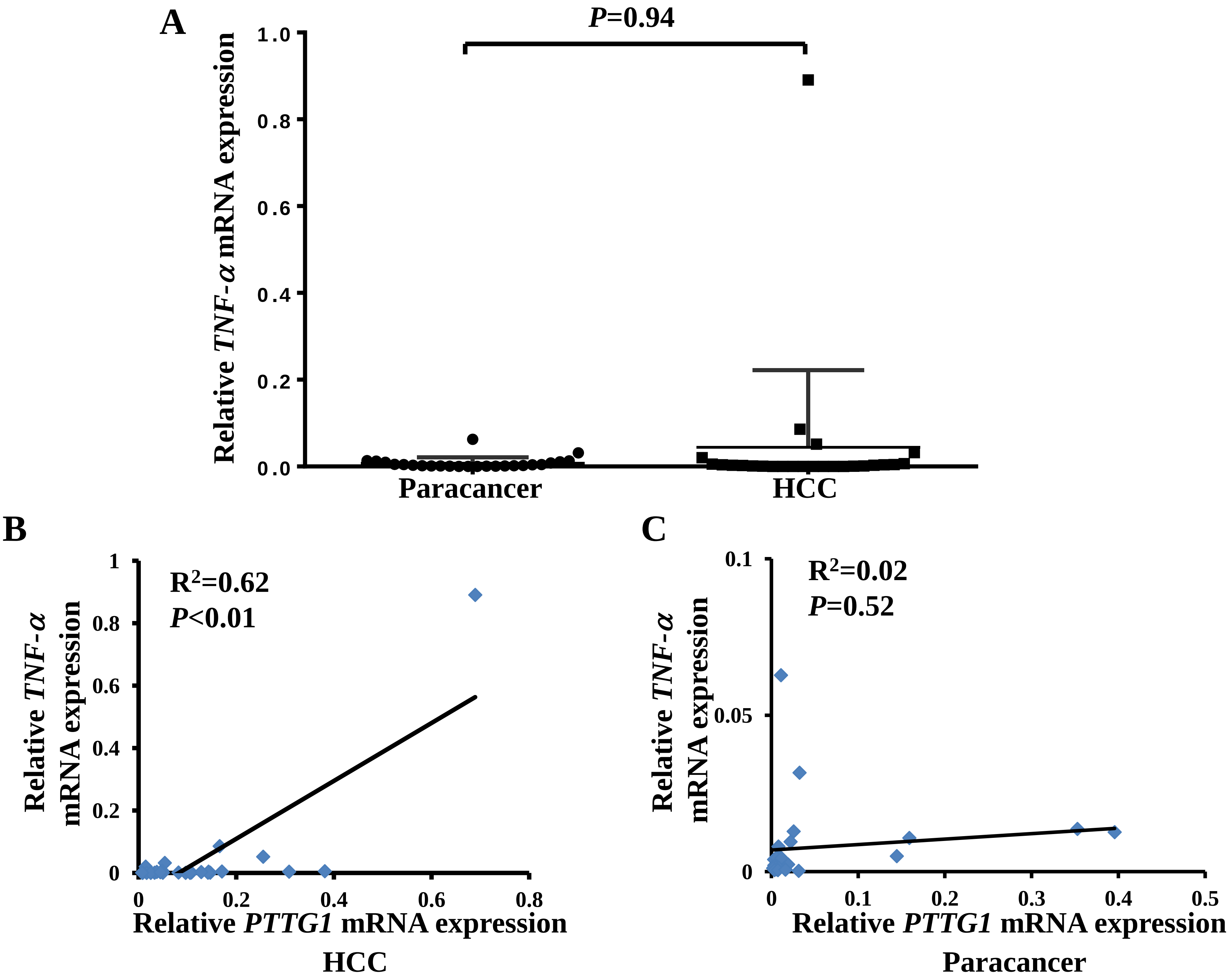
A
P=0.94
Relative TNF-α mRNA expression
1.0
0.8
0.6
0.4
0.2
0.0
Paracancer
HCC
B
R²=0.62
P<0.01
Relative TNF-α mRNA expression
1
0.8
0.6
0.4
0.2
0
0
0.2
0.4
0.6
0.8
Relative PTTG1 mRNA expression
HCC
C
R²=0.02
P=0.52
Relative TNF-α mRNA expression
0.1
0.05
0
0
0.1
0.2
0.3
0.4
0.5
Relative PTTG1 mRNA expression
Paracancer

Figure S1 *TNF-α* expression was determined in HCC and paired paracancer tissues. (A) *TNF-α* mRNA levels in HCC and its paracancer tissue were determined by real-time PCR. (B) Correlation of *TNF-α* with *PTTG1* mRNA expression in HCC tissues. (C) Correlation of *TNF-α* with *PTTG1* mRNA expression in paracancer tissues. Values are the mean ± SD. (n = 35 for HCC and its paracancer group respectively). Student's *t*-test and *Pearson's* linear correlation coefficient were used .

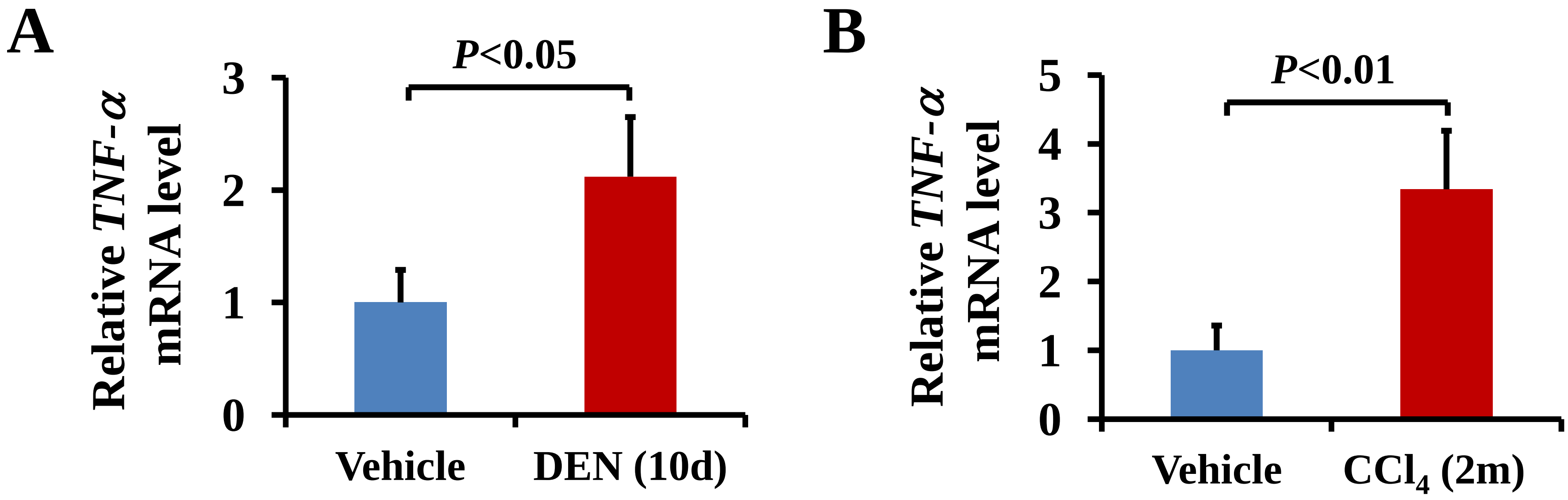

A
Relative TNF-α mRNA level
P<0.05
3
2
1
0
Vehicle
DEN (10d)
B
Relative TNF-α mRNA level
P<0.01
5
4
3
2
1
0
Vehicle
CCl4 (2m)

Figure S2 ***TNF-α*** **was upregulated in DEN- or CCl_4-treated mouse liver tissues.** (A) *TNF-α* mRNA level in DEN-treated mouse liver tissues was determined by real-time PCR. (B) After treated with CCl_4 for 2 months, *TNF-α* mRNA level in mouse liver tissues was determined by real-time PCR. Values are the mean $\pm$ SD. (n = 5 for each group respectively). Student's *t*-test.

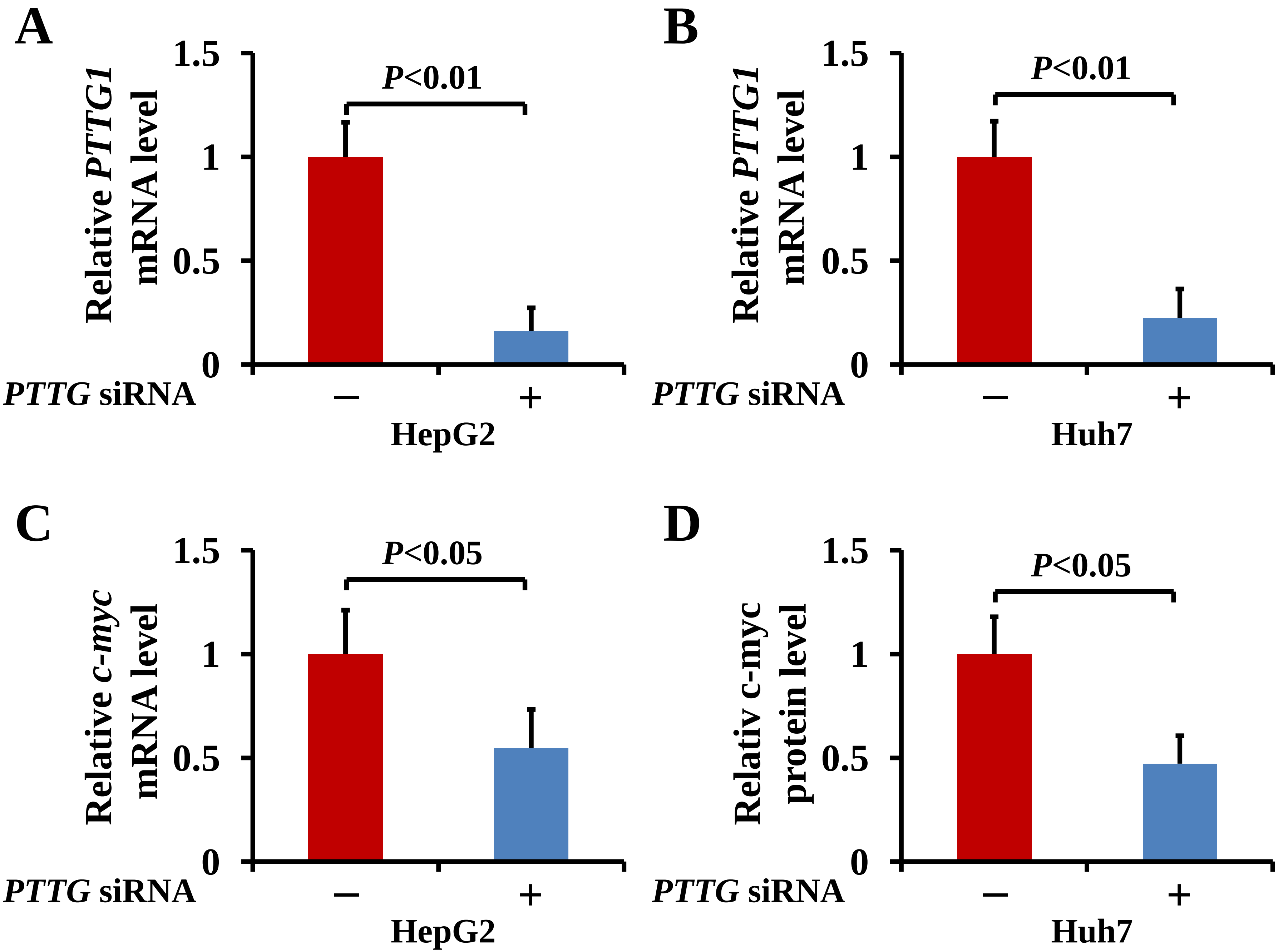
A
Relative PTTG1 mRNA level
1.5
1
0.5
0
P<0.01
PTTG siRNA
−
+
HepG2
B
Relative PTTG1 mRNA level
1.5
1
0.5
0
P<0.01
PTTG siRNA
−
+
Huh7
C
Relative c-myc mRNA level
1.5
1
0.5
0
P<0.05
PTTG siRNA
−
+
HepG2
D
Relativ c-myc protein level
1.5
1
0.5
0
P<0.05
PTTG siRNA
−
+
Huh7

Figure S3 *PTTG1* downregulation by siRNA reduced *c-myc* mRNA expression. (A, B) After *PTTG1*-siRNA administration for 48h in HepG2 and Huh7 cells respectively, *PTTG1* mRNA expression was downregulated by *PTTG1*-siRNA. (C, D) *PTTG1* downregulation by siRNA significantly reduced *c*-*myc* mRNA expression in HepG2 and Huh7 cells. The experiment was repeated three times. All values are the mean ± SD. Student's *t*-test.

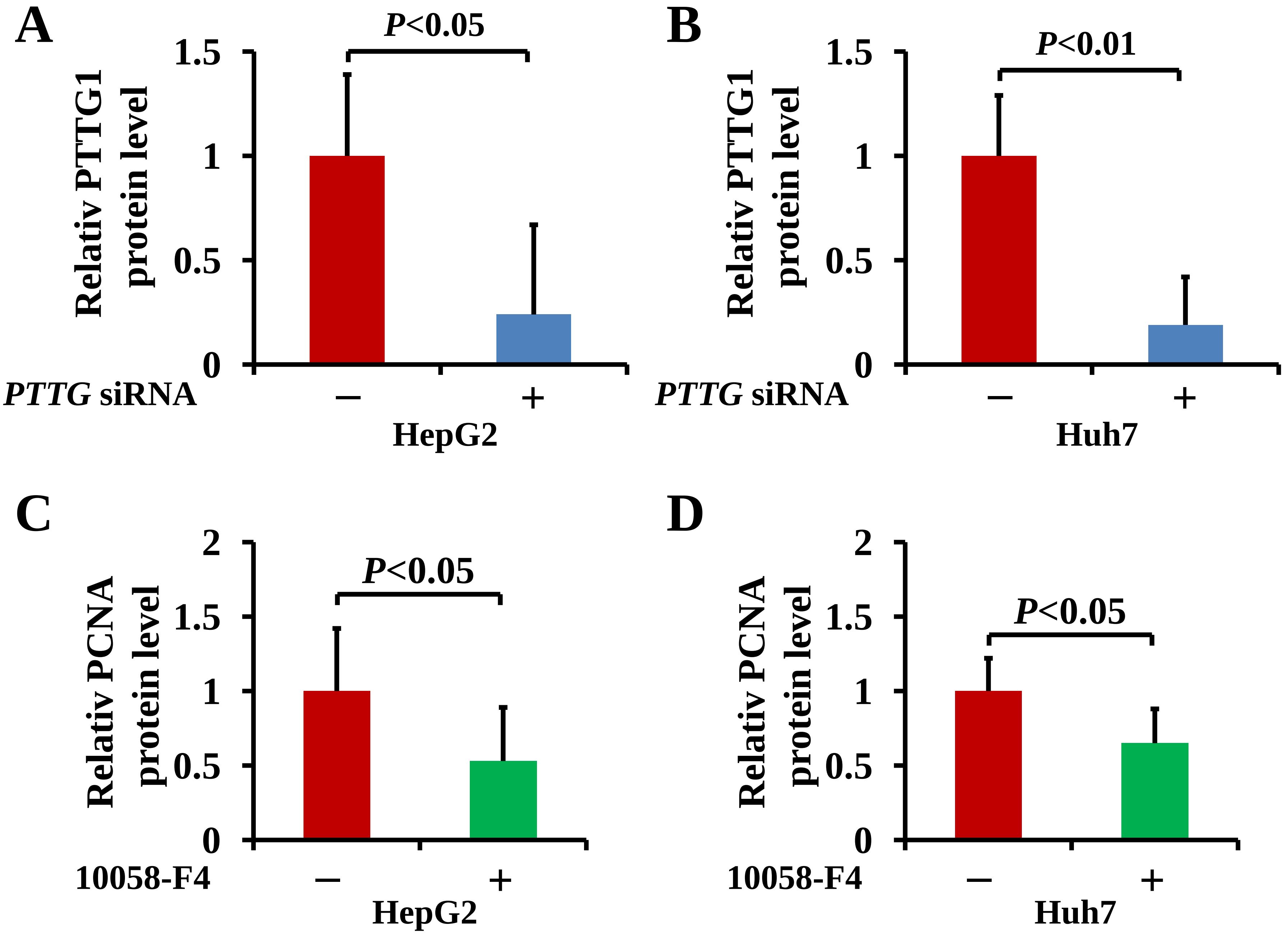

A
P<0.05
1.5
1
0.5
0
Relativ PTTG1 protein level
PTTG siRNA
−
+
HepG2
B
P<0.01
1.5
1
0.5
0
Relativ PTTG1 protein level
PTTG siRNA
−
+
Huh7
C
P<0.05
2
1.5
1
0.5
0
Relativ PCNA protein level
10058-F4
−
+
HepG2
D
P<0.05
2
1.5
1
0.5
0
Relativ PCNA protein level
10058-F4
−
+
Huh7

Figure S4 c-myc is an important target of PTTG1. (A, B) After *PTTG1*-siRNA administration for 48h, HepG2 and Huh7 cells were treated with TNF-α (20 ng/ml) for 8h. *PTTG1* expression was downregulated by *PTTG1*-siRNA. (C, D) HepG2 and Huh7 cells were treated by c-myc inhibitor 10058-F4 after TNF-α (20 ng/ml) administration. c-myc inhibitor repressed PCNA expression. The experiment was repeated three times. All values are the mean ± SD. Student's *t*-test was used.
